# Experience Playing a Musical Instrument and Overnight Sleep Enhance Performance on a Sequential Typing Task

**DOI:** 10.1371/journal.pone.0159608

**Published:** 2016-07-29

**Authors:** Matthew A. Tucker, Nam Nguyen, Robert Stickgold

**Affiliations:** 1 Center for Sleep and Cognition, Beth Israel Deaconess Medical Center, Boston, MA, United States of America; 2 Harvard University, Cambridge, MA, United States of America; Baycrest Health Sciences, CANADA

## Abstract

The smooth, coordinated fine motor movements required to play a musical instrument are not only highly valued in our society; they also predict academic success in areas that generalize beyond the motor domain, including reading and math readiness, and verbal abilities. Interestingly, motor skills that overlap with those required to play a musical instrument (e.g., sequential finger tapping) markedly improve (get faster) over a night of sleep, but not after a day spent awake. Here we studied whether individuals who play musical instruments that require fine finger motor skill are better able to learn and consolidate a simple motor skill task compared to those who do not play an instrument, and whether sleep-specific motor skill benefits interact with those imparted by musical experience. We used the motor sequence task (MST), which taps into a core skill learned and used by musicians, namely, the repetition of learned sequences of key presses. Not surprisingly, we found that musicians were faster than non-musicians throughout the learning session, typing more correct sequences per 30-sec trial. In the 12hrs that followed learning we found that sleep and musical experience both led to greater improvement in performance. Surprisingly, musicians retested after a day of wake performed slightly better than non-musicians who had slept between training and retest, suggesting that musicians have the capacity to consolidate a motor skill across waking hours, while non-musicians appear to lack this capacity. These findings suggest that the musically trained brain is optimized for motor skill consolidation across both wake and sleep, and that sleep may simply promote a more effective use of this machinery. In sum, there may be something special about musicians, perhaps a neurophysiological advantage, that leads to both the expected—greater motor speed at learning—and the surprising—greater motor skill improvement over time.

## Introduction

The use of fine motor skills so pervades our daily lives that their significance usually goes unnoticed. From the manipulation of small objects (e.g., fastening a button) to typing a term paper to playing a musical instrument, proper motor skill development is not only desirable, but necessary for optimal function. Not surprisingly, didactic methods for improving fine motor skill during early development have become ever more popular, especially in light of recent research linking fine motor skill development to cognitive abilities that generalize beyond the motor domain (e.g., math and reading readiness, and verbal ability) [1–3]. Additionally, brain plasticity and volumetric structure are impacted in individuals who have well-developed fine motor skills. In adult musicians, for example, highly skilled musicians show increased gray matter volume in brain regions associated with music reception and production (motor, auditory, and visuo-spatial cortices) compared to less-skilled musicians and non-musicians [4]. Importantly, these structural differences observed in adult musicians are to an extent learning-induced, as opposed to purely innate capabilities. This has been demonstrated in a study of children who completed a 15-month music training program. Children who completed the program showed increased volume in motor and auditory cortex, and the corpus callosum, compared to children who did not participate in the training program [5]. These structural differences are paralleled by greater functional connectivity in musicians between and within hemispheres in brain regions associated with musical expression, including sensorimotor, prefrontal (Broca’s area), and auditory cortices [6].

Given the importance of developing fine motor skill abilities, much research has been devoted to understanding the factors that enhance the acquisition and retention motor skills. One fruitful body of research has demonstrated numerous times that simply getting a good night of sleep imparts a dramatic boost to motor performance. Specifically, if a healthy young individual learns a task requiring fine motor skill (e.g., a repetitive typing task) and is retested on the same task following an interval of sleep, performance (typing speed) significantly improves relative to their baseline performance, and this improvement is significantly greater than observed in individuals who remain awake prior to retest [7].

One task that assesses the impact of sleep on this type of fine motor skill is the motor sequence task (MST), which requires the subject to use the four fingers of their non-dominant hand to type a 5-digit sequence of numbers as fast and accurately as possible [7]. Many studies over the past decade demonstrate just how important sleep is for enhancing motor skill performance [8–11]. For example, if a subject, after initial MST training, is given extra practice across the day (2 trials every 4 hours), performance will improve 1–2% with each practice session. However, if the same subject then sleeps, performance increases an additional 17% beyond what was achieved with extra practice [12]. If subjects are given two retest opportunities, one following a day of wake, and the other following a subsequent night of sleep, sleep imparts a significant over-sleep benefit above that observed following a non-significant change after wake alone. The same sleep-wake difference is observed when the sleep period occurs first and wake period second, implicating sleep as a major factor in the promotion of fine motor ability. These same motor skill benefits of overnight sleep have also been observed following daytime naps [13–15] or shorter intervals (~3.5hrs) of nocturnal sleep [11] and, importantly, have been linked to sleep-specific physiology. For example, several studies reveal that greater amounts of stage 2 sleep enhance motor skill performance [11, 13], and that increased number and density of sleep spindles, a physiological signature of stage 2 sleep, promote greater over-sleep gains in motor skill performance [13, 16–18].

In most studies that examine MST performance in healthy young college students, potentially important differences in prior motor skill ability have not been taken into account. One might expect that individuals with well-developed fine motor skills would be better equipped to learn a simple motor task than subjects who are less skilled. Previous research suggests that this may be the case, demonstrating superior acquisition of a sequence typing task in pianists v. non-pianists [19]. Concerning motor memory consolidation in those with music experience, it has been shown that musicians demonstrate significant over-sleep gains on a sequence-typing task [20]. What is less clear, however, is the degree to which musicians may better consolidate the skill (become faster) during a 12-hr offline interval compared those without experience playing a musical instrument, and whether sleeping during this interval will modulate the effects observed with musical experience alone. The present study examines the effect of preexisting fine motor skill experience (playing a musical instrument that requires fine motor skill) on MST performance across a 12hr day of wakefulness or a 12hr interval containing a night of sleep.

## Methods and Materials

### Subjects

Forty right-handed undergraduate students from Boston area universities participated in the study (26 females, mean age = 21.1 ± 3.2yrs). In this 2x2 Music/No-Music by Sleep/Wake between subjects design, Music and No-Music subjects were randomly assigned to the sleep or wake condition. Subjects in the Music groups reported playing at least one instrument that required fine motor coordination of the fingers (i.e., that required skill with key presses similar to that of the motor sequence task (MST)). Examples of instruments that were allowed in this study were: piano (n = 9), flute (n = 6), alto saxophone (n = 1), violin (n = 5), and guitar (n = 2). Each of these instruments requires the development of individual fine finger movements of the left (non-dominant) hand. Only one subject from the original music sample, a trombone player, was excluded from analysis based on this criterion, as playing trombone does not require the use of fine finger movements. The final group composition was as follows: Sleep-Music (n = 10), Wake-Music (n = 10), Sleep-NoMusic (n = 11), Wake-NoMusic (n = 9). Subjects were instructed to maintain a regular sleep schedule, confirmed at the time of the study by a 3-day retrospective sleep log. Subjects abstained from caffeine and alcohol 24hrs prior to, and for the duration of, the study. Subjects in the wake condition were instructed to not nap prior to the retest session. Subjects were excluded from data analysis if they were taking medications known to interfere with sleep or cognitive function. All subjects provided written consent to participate in the study, which was approved by the Beth Israel Deaconess Medical Center Institutional Review Board.

### Motor Sequence Task

The MST is a standard test of fine motor skill and memory that has been used in a number of previous studies [7, 11, 21, 22]. During this task, subjects are instructed to repeatedly type a 5-digit sequence of numbers (4-1-3-2-4) with their left (non-dominant) hand “as quickly and accurately as possible” for each of twelve 30sec trials. The 5-digit number is always displayed at the top of the screen, and each keystroke is represented by the appearance of a dot on the screen starting from the left edge of the monitor. Subjects are given a 30sec rest period between trials. Only the number keys at the top left of the keyboard were used. Subjects complete 12 trials during the training session and 12 trials at retest, with number of correctly typed sequences per trial used as the primary dependent measure. For each of the four groups we examined (*i*) Trial 1 performance (number of completed sequences on the first 30sec training trial), (*ii*) Final training performance (average number of completed sequences on the last 3 training trials), (*iii*) Change in performance across the training session (Final training performance—Trial 1 performance) (*iv*) Improvement from training to retest (first 3 retest trials—last 3 training trials) and (*v*) change from the beginning of retest to the end of retest (last 3 retest trials—first 3 retest trials). Improvement measures were calculated as percent change.

### Procedure

Subjects arrived at a computer laboratory on the Harvard University campus at either 9am (Wake subjects) or 9pm (Sleep subjects). They completed a 3-day retrospective sleep log to assess sleep regularity and quality. The Epworth Sleepiness Scale [23] was administered to assess general sleepiness. Prior to task training subjects completed two visual analog scales, one asking about their ability to concentrate and the other asking about how refreshed they feel. The Stanford Sleepiness Scale [24] was also used to assess state alertness/sleepiness.

Subjects trained on the MST (12 trials), and were retested 12hrs later (12 trials) after a day of wakefulness or a night of sleep. Following the retest session subjects completed an exit questionnaire that asked whether the subject played a musical instrument and, if so, to list the instrument(s).

## Results

### Sleep Quality and Alertness

Sleep quality and alertness measures did not differ across the four groups unless noted: Retrospective 3-Day Sleep Log: average bedtimes (Range: 12:17am-1:20am, sleep-music (1:20am ± 23min) v. sleep-no music (12:17am ± 20min), p = 0.04), sleep duration (Range: 7.7–8.2 hrs) and time to fall asleep at bedtime (Range: 9.7–15.8 minutes); Stanford Sleepiness Scale (SSS) scores at training (Range: 2.0–2.6); Epworth Sleepiness Scale (ESS) scores (Range: 5.8–9.1 wake music (9.1 ± 1.0) > sleep-no music (5.8 ± 1.0), p = 0.03); Visual analog scales (VAS) for subjective ‘ability to concentrate’ (all p values > 0.05), ‘feeling refreshed’ (wake-music (86.6 ± 6.6) > sleep-no-music (60.2 ± 8.5), p = 0.02), and reported motivation to do well on the task (all p values > 0.05).

### Training Performance

**Fig 1:**
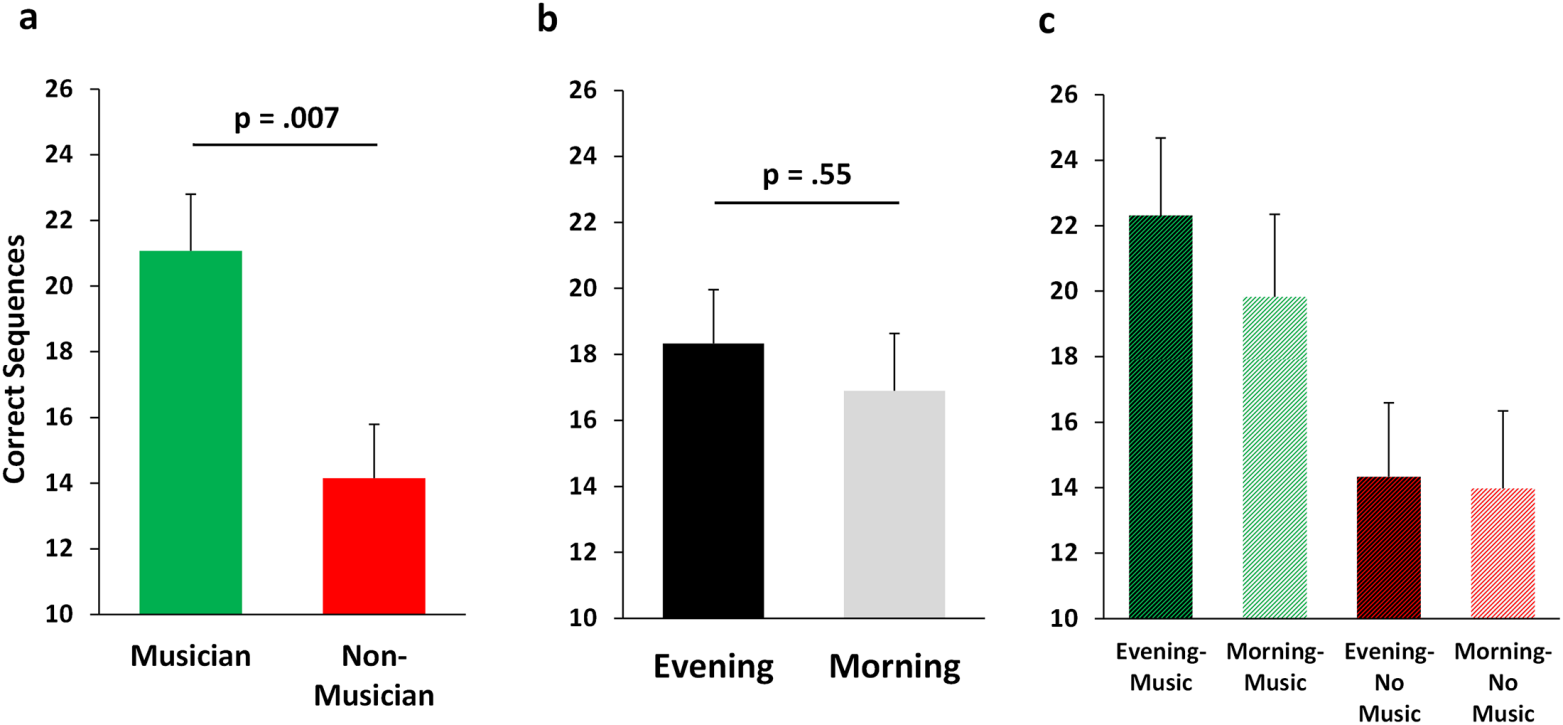
Performance at start of training. On the first training trial, without any prior experience with the MST, musicians typed more correct sequences than non-musicians (a), but the time of training (evening v. morning) did not impact performance (b). Performance for each of the four groups is plotted (c).

**Fig 2:**
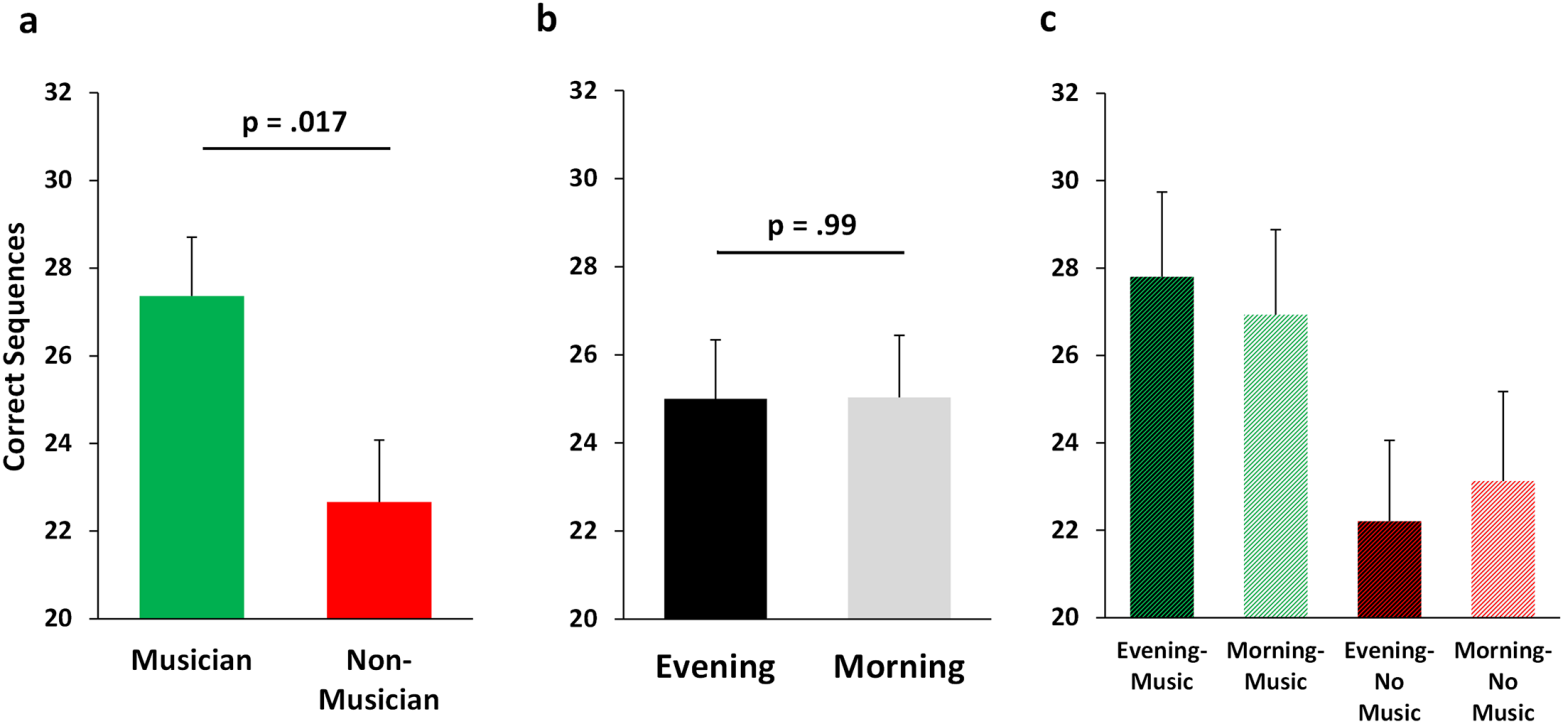
Performance at the end of training. Similar to trial 1 performance, musicians continued to outperform non-musicians (a), with non-musicians typing a similar number of sequences as has been observed in previous MST research. Time of day of MST training did not impact the number of correct sequences typed during the training session (b). Performance for each of the four groups is plotted (c).

Musicians were faster (typed more correct sequences) than non-musicians on the first MST trial of the training session (music: 21.1 ± 1.7 (SEM); no-music: 14.2 ± 1.6; F_3,32_ = 8.43, p = 0.007; Fig 1a), achieving a score comparable to scores a typical subject achieves only by the end of the MST training session [11, 12]. However, there was no main effect of training in the evening (sleep group) vs. the morning (wake group) for MST Trial 1 performance (18.3 ± 1.6 vs. 16.9 ± 1.7, F_3,36_ = 0.36, p = 0.55; Fig 1b), and no interaction effect (p = 0.66; Fig 1c). Musicians continued to perform better than non-musicians across the MST training session, finishing the training session (Trials10-12) typing 4.7 (21%) more correct sequences per trial than non-musicians (27.4 ± 1.6 v. 22.6 ± 1.0, t_38_ = 2.50, p = 0.017; Fig 2a). However, the amount of improvement over the training session was similar for musicians (6.4 ± 1.2 sequences) and non-musicians (8.8 ± 1.1 sequences; F_3,32_ = 2.50, p = 0.12), as well as in the evening (7.1 ± 1.2 sequences) and morning (8.1 ± 1.2 sequences; F_3,32_ = 0.39, p = 0.54). Time of training also did not affect MST training final performance (Trials10-12 –morning: 25.1 ± 1.1, evening: 24.9 ± 1.7 sequences; F_3,36_ = 0.00, p = 0.99; Fig 2b), suggesting negligible influence of circadian biology on MST performance and learning. There was no interaction between evening/morning and music/no-music on training performance (p = 0.65; Fig 2c).

### Motor Skill Improvement across Sleep and Wake in Musicians and Non-Musicians

**Fig 3:**
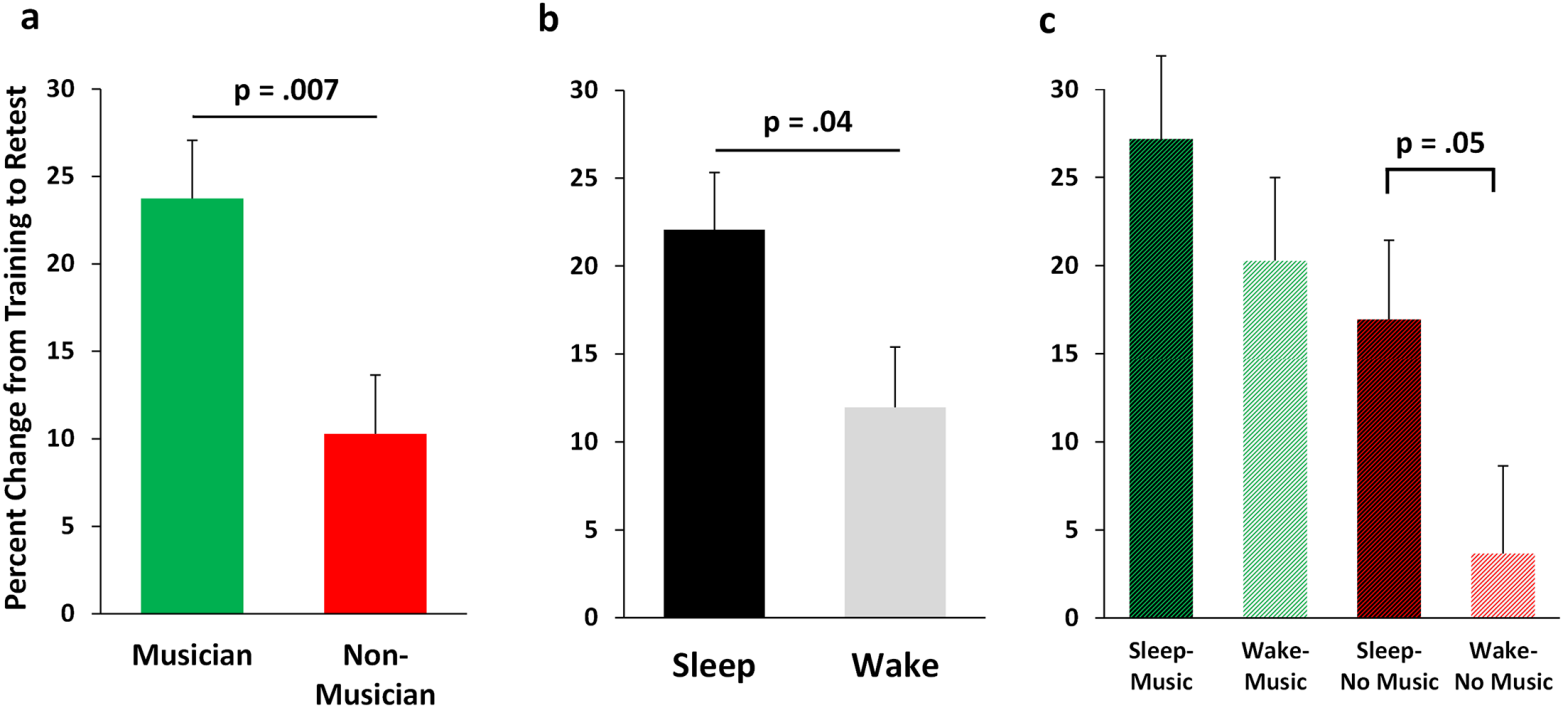
MST improvement. Percent increase in MST speed from training to retest after a night of sleep or a day of wake (a), in musicians and non-musicians (b), and across all groups (c). The significant difference between the Sleep and Wake (No-Music) group shows the typical difference observed in studies of the impact of sleep on MST performance (~20% v. 4% increase in performance after sleep and wake, respectively).

**Fig 4:**
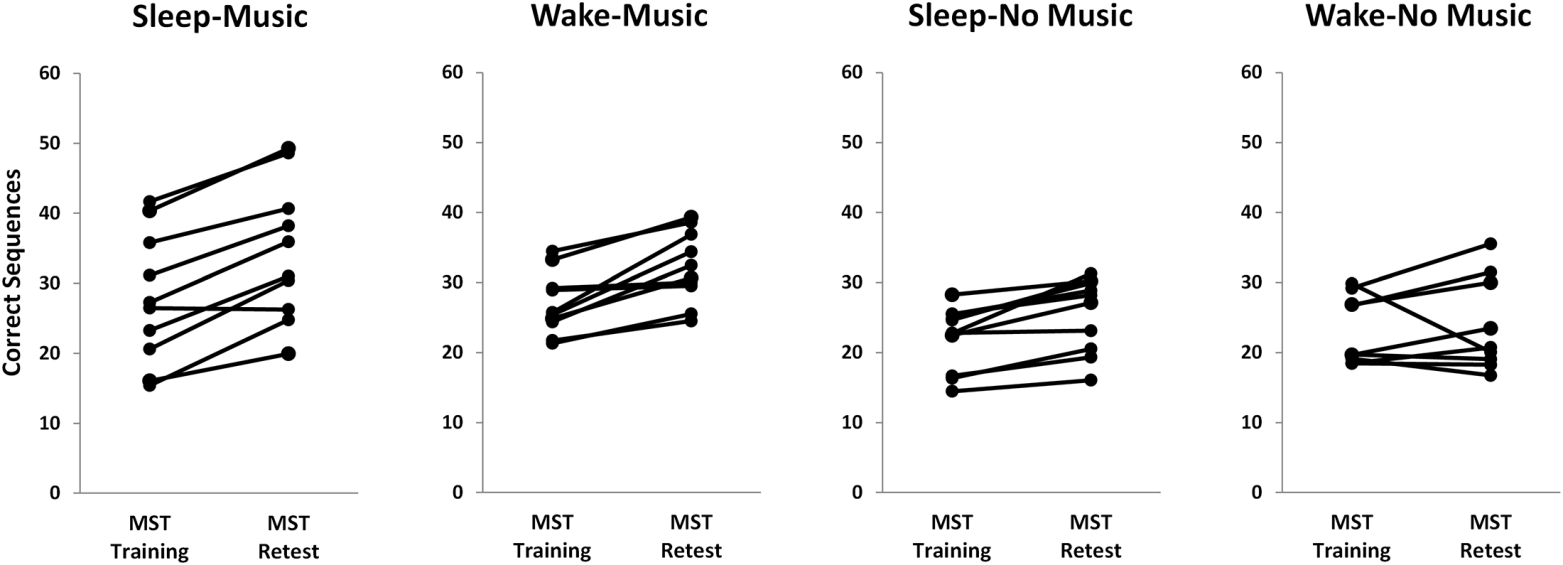
Individual subject performance at MST training and retest. MST training is measured as the average number of correctly typed sequences across the last 3 training trials (10–12); MST retest represents the average number of correctly typed sequences across the first 3 retest trials (1–3).

We found main effects of sleep condition and music training on MST improvement from training to retest (Fig 3; see Fig 4 for individual subject data). Those who slept improved more than those that were awake during the 12hr training-retest interval (over-sleep improvement: 21.8 ± 3.2%; over-wake improvement: 12.4 ± 4.0%, F_3,36_ = 4.56, p = 0.04; Fig 3a). Subjects with music training improved more than those without music training, even calculated as a percent increase in performance (musician improvement: 23.7 ± 3.5%; non-musician improvement: 11.0 ± 3.4%, F_3,36_ = 8.07, p = 0.007; Fig 3b). However, there was no music x sleep condition interaction (p = 0.51; Fig 3c). Sleep and Music both benefitted offline improvement, with the Sleep/Music group showing the most offline improvement, and the Wake/No-Music group showing the least improvement (p = 0.002, LSD-corrected; Fig 3c). The correlation between training performance and improvement was not significant (whole sample: r = 0.04, p = 0.81, all group p values: p > .21), suggesting that how well a subject performs at training (not sleep or music experience) is not a predictor offline MST consolidation.

When analyzing the individual groups, we found that the Sleep-No Music group improved by 17% (t_10_ = 5.64, p = 0.0002), whereas the Wake-No Music group improved by a non-significant 3.7% (t_8_ = 0.61, p = 0.56), which is in keeping with many previous findings using the MST. The difference between these two groups was significant (t_18_ = 2.10, p = 0.05). However, the difference between the Sleep-Music and Wake-Music group did not reach significance (p = 0.34). Finally, an unexpected outcome, which should be noted, is that the Wake-Music group improved just as much as the Sleep-No Music group (20% v. 17%, respectively; F_1,19_ = 1.10, p = 0.53; Fig 3c), demonstrating that prior musical training alone is associated with offline daytime wake improvement equivalent to the typically observed overnight sleep-dependent improvement.

### Change in Performance across the Retest Session

**Fig 5:**
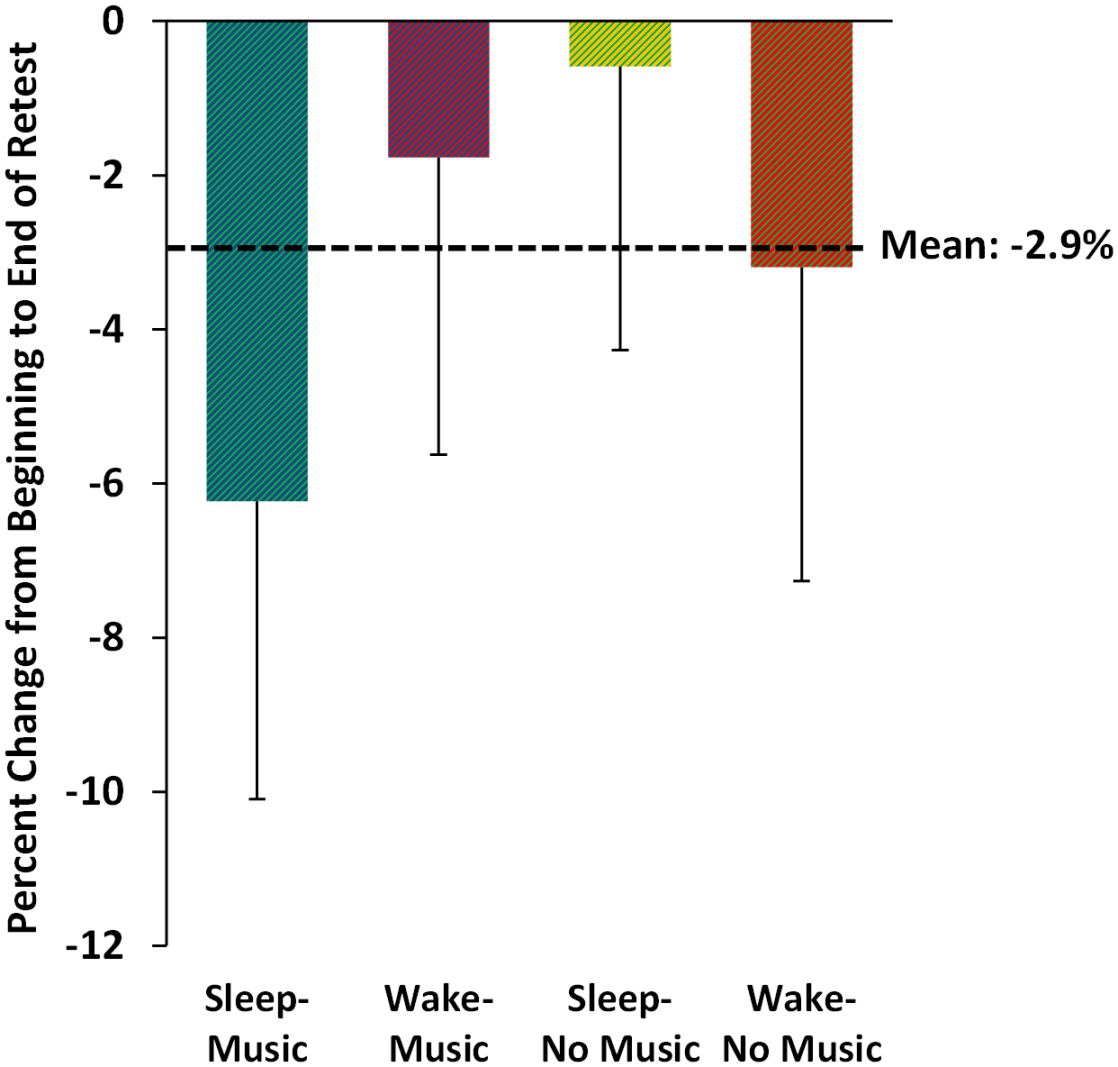
Change in MST typing speed across the retest session. All groups demonstrated a non-significant decrease in performance across the 12 retest trials; all group comparisons, and percent change for each individual group, were non-significant.

Finally, to assess whether groups sustained their performance across the 12-trial retest session, we compared the first 3 retest trials (trials 1–3) to the last 3 retest trials (trials 10–12) in each group. All groups’ performance declined modestly and non-significantly across the retest session (-2.88 ± 1.88%, p = 0.13; Fig 5), and no individual group demonstrated a significant decline (one-sample t-tests, all p-values > 0.17). Additionally, there were no differences across groups (ANOVA, F_3,36_ = 0.27, p = 0.85), and there were no main effects of sleep (p = 0.81) or music condition (p = 0.59).

## Discussion

Fine motor skill is crucially important in our daily lives, and recent research confirms that well-developed fine motor skills in children predict school readiness, academic achievement, and enhanced reading and math ability. On a physiological level, the skills acquired by learning how to play an instrument are related to enhanced brain plasticity and greater interconnectivity between a wide-ranging network of brain regions, including auditory, sensorimotor, and prefrontal cortices.

In this study we found that subjects who reported experience playing a musical instrument requiring fine finger motor skills demonstrate two important benefits regarding motor skill performance. Not surprisingly, this type of experience enables subjects to perform a motor skill task better than non-musicians during the initial task acquisition phase. However, in addition to being significantly faster on the task from beginning to end of the learning session, musicians also demonstrated significant improvement (above training) at retest 12 hours later, regardless of whether they had slept or stayed awake. Our initial belief was that the degree of improvement (percentage increase in speed) between musicians and non-musicians would be more similar because musicians would be starting from a higher baseline speed at training, possibly leaving them less room for improvement over the ensuing 12-hr interval. This was not the case; individuals who play a musical instrument got significantly faster on the motor task than non-musicians, regardless of whether they slept or stayed awake between training and retest. In other words, their musical experience imparted a double benefit: a learning *and* consolidation benefit.

The findings regarding musical experience were paralleled by our sleep findings. As in past studies [7, 12], we did not see a difference in training performance dependent on whether subjects trained after sleep (in the morning) or wake (in the evening), but we did see a significant motor skill improvement over a night of sleep compared to a day of wake, in musicians *and* non-musicians. This over-sleep benefit is in line with numerous past studies of sleep and motor skill enhancement [25]. Interestingly, there was no interaction between music experience and sleep/wake condition in this study, suggesting that sleep and musical experience are separate factors that each contribute to the consolidation of motor skill.

As a corollary to this general benefit of music experience on motor skill performance, we found that musicians improved as much on the MST over a day of wake as did non-musicians over a night of sleep. While past studies have largely demonstrated a non-significant performance improvement on the MST over a day of wake, these findings suggest the possibility that offline consolidation of the MST can occur in the waking brain under certain conditions (i.e., when equipped with past experience playing a musical instrument), and that the benefits can be equal to those following a night of sleep.

As a point of interest, it is worth noting that the sleep findings of this study parallel those of a previous study from our lab [26], a study showing that 1) sleep (as opposed to wake) benefited memory consolidation, and 2) that if subjects were rewarded for their performance ($1 for each correct answer) they performed better than if they were not rewarded. In that study, sleep and reward each contributed to enhanced memory consolidation but did not interact with each other. It will be interesting to see whether this is a consistent finding in future research, one suggesting that sleep represents a “rising tide that lifts all boats,” a biological state that does not, for example, depend on whether the subject is a musician, or if performance is rewarded or not.

While the findings of this study provide an initial understanding of how sleep and music experience contribute to motor skill consolidation, future research should aim to more carefully characterize musical ability. One limitation of this study was that we simply asked subjects whether they played an instrument and, if so, which instrument(s), to determine if they employed fine finger movements akin to those used in the MST. However, we did not collect information regarding the extent of subjects’ expertise with each instrument, or how long it had been since they last played the instrument. It will be of interest in future studies to more closely examine whether there is a relationship between expertise, instrument(s) played, and their impact on memory processing. A second limitation of the study was the small sample size. While the individual group sample sizes were similar to past studies assessing the impact of sleep on MST performance [7, 12], a larger sample size would allay concerns about adequate statistical power. Also, while we did not collect sleep recordings in this study, it is exciting to speculate about the possibility that sleep-specific physiological characteristics differentiate musicians from non-musicians. There is now ample evidence demonstrating a role for stage 2 sleep spindles, and time spent in stage 2 sleep, in the processing of motor skills [7, 11, 13, 16, 27]. Might musicians express increased spindle activity, or spend more time in stage 2 sleep? Are these increases innate, or can they be obtained through increased experience playing an instrument? Hopefully, future research on music experience and sleep will provide compelling answers to these interesting questions.
